# The Rate of Decrease in Brain Perfusion in Progressive Supranuclear Palsy and Corticobasal Syndrome May Be Impacted by Glycemic Variability—A Pilot Study

**DOI:** 10.3389/fneur.2021.767480

**Published:** 2021-11-08

**Authors:** Piotr Alster, Anna Dunalska, Bartosz Migda, Natalia Madetko, Leszek Królicki

**Affiliations:** ^1^Department of Neurology, Medical University of Warsaw, Warsaw, Poland; ^2^Students' Scientific Circle of the Department of Neurology, Medical University of Warsaw, Warsaw, Poland; ^3^Diagnostic Ultrasound Lab, Department of Pediatric Radiology, Medical Faculty, Medical University of Warsaw, Warsaw, Poland; ^4^Department of Nuclear Medicine, University Clinical Center, Medical University of Warsaw, Warsaw, Poland

**Keywords:** PSP, CBS, SPECT, prediabetes, perfusion

## Abstract

Progressive supranuclear palsy (PSP) and corticobasal syndrome (CBS) are tauopathic parkinsonian syndromes, presently lacking disease-modifying treatments. Patients affected by these diseases suffer due to multidimensional deteriorations resulting in motor and cognitive impairment. Previously published research has confirmed risk factors that may impact the course of PSP and CBS, among them hypertension and diabetes. Less data is available regarding prediabetes and glycemic variability. In this study, 26 patients with clinical diagnoses of PSP and CBS were examined using glycated hemoglobin and perfusion single-photon emission tomography (SPECT). Patients were divided into two groups—PSP/CBS patients with glycated hemoglobin (HbA1c) below and above 5.7%. The results of the perfusion evaluation were compared with the values from healthy volunteers from the software's database. A decrease in perfusion in certain regions of interest was observed among patients affected by increased glycemic variability. A more pronounced decrement in perfusion was observed only in some regions of interest—the hippocampus, pons, left thalamus, right insula. The results indicated that, among PSP/CBS patients, individuals with more pronounced glycemic variability had more severe hypoperfusion in certain brain regions in comparison with PSP/CBS patients without carbohydrate metabolism disorders. Due to the fact that PSP and CBS are associated with cognitive impairment, an additional decrease in perfusion in the hippocampal area may impact the rate of cognitive deterioration.

## Introduction

### PSP and CBS

Progressive supranuclear palsy (PSP) and corticobasal syndrome (CBS) are the most common clinical manifestations of four-repeat tauopathies (1–3). The clinical syndrome largely lacks clinicopathologic correlation with progressive supranuclear palsy (PSP) pathology and CBS lacks correlation with corticobasal degeneration (CBD). The correlation of syndrome and pathology in the most common variant of PSP—Richardson's syndrome—is estimated at ~90%. However, in CBS, up to half of the cases are not related to CBD pathology (4). Among patients with longer disease duration, clinical manifestations of PSP and CBS overlap. Additionally, PSP may be a clinical manifestation of CBD pathology and, less commonly, CBS may be a clinical manifestation of PSP pathology. There is growing interest in the issues related to diagnosing PSP-CBS or four-repeat tauopathy (5). Although the boundaries between the pathologies of PSP and CBD related to tufted astrocytes and astrocytic plaques are not in question, the differentiation between their clinical manifestations is less obvious (4). Several studies highlight the questionable significance of PSP and CBS differentiation, especially in advanced stages (4, 6). The diseases share similar risk factors as hypertension and diabetes, however, the exact mechanism of their correlation has not been explored (7, 8). In PSP, both hypertension and diabetes were identified as risk factors (8). In PSP and CBS, some of the clinical manifestations are based on vascular pathogenesis (9, 10). Regarding the lack of effective disease-modifying treatments for PSP and CBS, multiple questions are raised in the context of modifiable factors. The issue of diabetes and hypertension is evaluated and studied in the context of various neurodegenerative disorders, less attention is focused on preceding conditions such as prediabetes or glycemic variability, where insufficient glucose values do not meet the criteria for diabetes.

### Glycemic Variability in Brain Diseases

There is little data available concerning the impact of glycemic variability on the central nervous system. Reoccurring hypoglycemia may exacerbate oxidative stress and inflammation, leading to damage to vulnerable brain regions and the acceleration of cognitive decline (11). Glucose variability is the major predictor of cerebral vasomotor reactivity reduction induced by acute hyperglycemia, which may be one of the early mechanisms of cerebrovascular damage in diabetes mellitus (DM) (12). Dysregulation of glycemic levels, recurring over time, might contribute to brain atrophy and cognitive decline in individuals with type 2 diabetes (DM2) (13). Glycemic variability and hyperglycemia during acute ischemic stroke are correlated with a higher risk of post-stroke cognitive impairment and early neurological deterioration (14, 15). Moreover, it was shown that, among patients with DM2 with acute ischemic stroke, the initial glycemic variability increased cardiovascular events and was an independent predictor of death (16, 17). Variability in long-term glycemic control is also connected with a higher white matter hyperintensity load in elderly patients with DM2 (18). In patients with traumatic brain injury, variable glucose blood levels were significantly associated with a worse long-term functional outcome (19).

## Materials and Methods

### Patients

The study included 26 patients (16 women and 10 men) aged 57 to 83 with possible clinical diagnoses of PSP and CBS made in accordance with the currently applicable criteria (1, 2). Disease duration varied from 4 to 6 years. Subjects evaluated in the study were affected by mild (MMSE = 19–23 points) or moderate cognitive impairment (MMSE = 10–18 points). Patients were affected by limited mobility and suffered due to multiple collapses. Examinations were performed by neurologists experienced in movement disorders. Each patient underwent a laboratory assessment for glycated hemoglobin level and a single-photon emission computed tomography (SPECT) examination of perfusion. For each patient, an evaluation of perfusion in 19 regions of interest was performed. All subjects included in the study were diagnosed with PSP or CBS and had levels of glycated hemoglobin lower than 6.5%. Each participant provided written informed consent. The following exclusion criteria were used in the study—history of stroke, vascular lesions with cognitive impairment, neoplasms, posttraumatic brain injury, autoimmune brain inflammation, or brain infection. Patients included in the study were divided into two groups: “A”-−12 patients with PSP/CBS without any abnormalities in the context of glucose level, and “B”−14 patients with PSP/CBS and significant glycemic variability. The level of glycated hemoglobin in group “A” varied from 4.9 to 5.6%. In “B” the levels varied from 5.7 to 6.4%. The perfusion of patients from groups “A” and “B” was compared with healthy volunteers from the software's database. Among all of the patients included in the study, fasting blood glucose level was below 100 mg/dl.

### SPECT

SPECT with Technetium-99m hexamethylpropyleneamine oxime (99mTc-HMPAO) as a radiotracer was performed in all patients. The patients were placed in a quiet, dimly lit room in a supine position. Seven hundred and forty MBq of ^99^mTc-HMPAO was administered. A dual-head gamma camera SPECT/CT (Symbia T6, Siemens Healthcare) with low energy high resolution (LEHR) parallel-hole collimator was used. Step and shoot acquisition mode was used and sequences of 128 frames on a 128 x 128 matrix were obtained (64 projections per head, 30 s per projection). The photopeak was set at 140 keV with 10% window on either side of the photopeak. All images were reconstructed with filtered back projection and smoothed with a filter. The reconstructed images were corrected for gamma-ray attenuation with a measured correction matrix obtained from a computed tomography scan.

Post-processing analysis was performed with Scenium software (Siemens Healthcare Medical Solutions USA, Inc.). The data were compared with a reference database comprised of 99mTcHMPAO brain scans of 20 healthy volunteers with an age range of 64–86 years (mixed population of women and men). Standard deviation values from the mean for each voxel were computed. The mean was taken from the corresponding voxel in the normal brain. Statistics were displayed on a voxel-by-voxel basis. The regions of interest (ROI) were predefined on a high-resolution T1 MRI volume scan. The SPECT and MRI data were merged. The mean standard deviation (SD) was calculated for each ROI. The SD of perfusion in the basal ganglia, frontal lobes, cerebellar hemispheres, and thalami were subsequently examined among all patients. The results were then analyzed using the Kruskal-Wallis test, which showed whether the differences in perfusion of the selected brain regions were statistically significant. The regions with significant differences were then additionally evaluated using *post-hoc* analysis.

The authors of the study decided to study CBF disorders in all regions of the brain, including areas that are not affected by the lesions. Correct values for the accumulation of 99mTc HM-PAO in these areas can be taken as evidence that the test method is methodologically correct. We used a professional program that also includes a verified database (control group). The control group was thoroughly examined by the manufacturer and corresponds to the age of the group of patients we studied. Performing check-ups with radiopharmaceuticals and SPECT-CT would be unethical due to exposure to ionizing radiation and the lack of availability of a professional, registered program for the analysis of test results.

### Laboratory Testing

Blood samples were collected from patients during their hospitalization in the Department of Neurology. Ethylenediaminetetraacetic acid (EDTA) was used to avoid extracorporeal coagulation before the analysis. Blood samples were analyzed automatically using high-pressure liquid chromatography in the Department of Laboratory Diagnostics of the Mazovian Bródno Hospital. The value of glycated hemoglobin was obtained automatically.

### Statistical Analyses

Data analyses were performed with the use of original statistical programs and available modules of the Statistica software (version 13.1 Dell. Inc. Statsoft). Continuous data were used for analyses. All results were expressed as means, with minimal and maximal values, and standard deviation with a 95% confidence interval. In subgroup analysis, we used the Mann-Whitney U test. Spearman correlation coefficient (Rs) was used for determining the relationship between glycated hemoglobin level and CNS perfusion. We first tried to apply the Benjamini-Hochberg method for reducing the false discovery rate (FDR) for multiple hypothesis testing, but unfortunately, no p-value survived after this correction. Thus, we chose to set a more restrictive, uncorrected significance threshold at *P* < 0.01 for multiple comparison correction.

## Results

**Figure 1:**
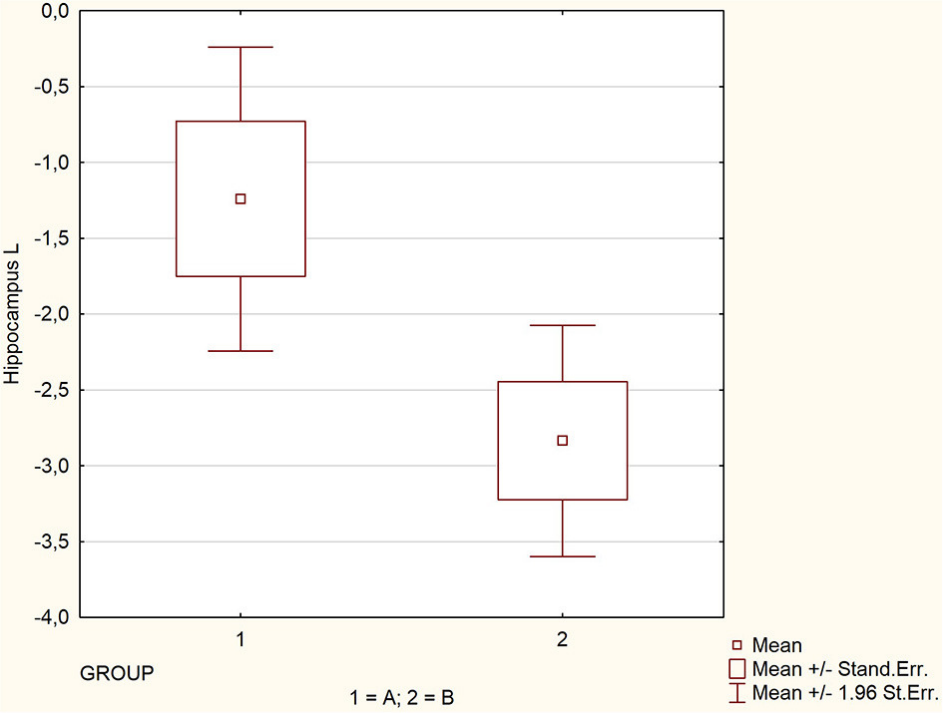
Comparison of perfusion in regions significantly affected by glycemic variability.

**Figure 2:**
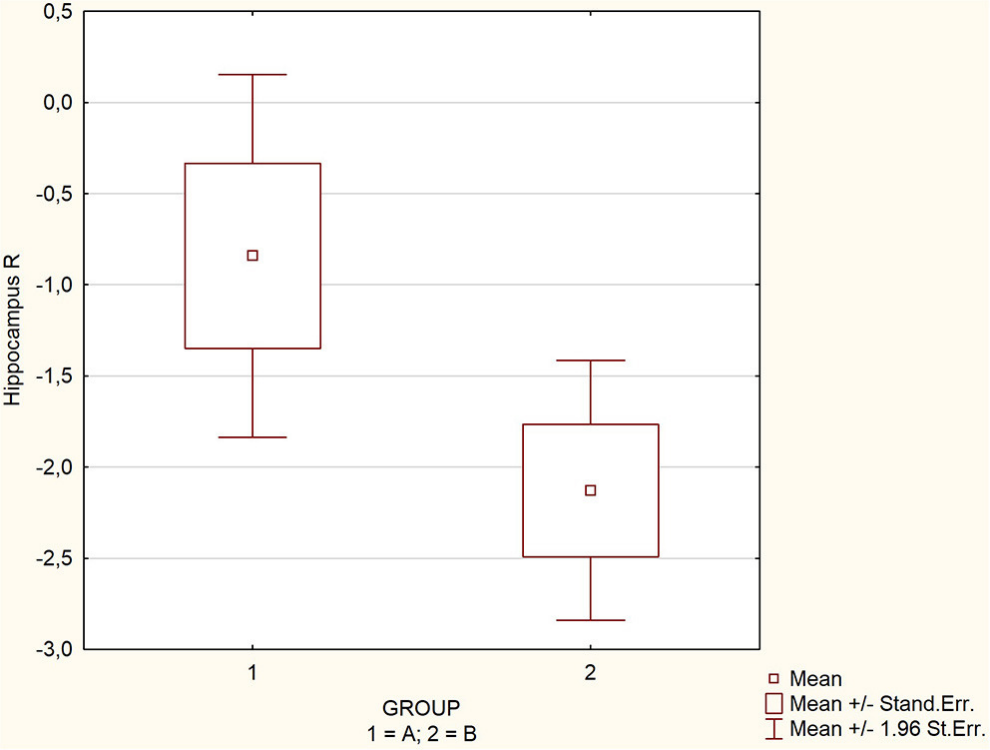
Comparison of perfusion in regions significantly affected by glycemic variability.

**Figure 3:**
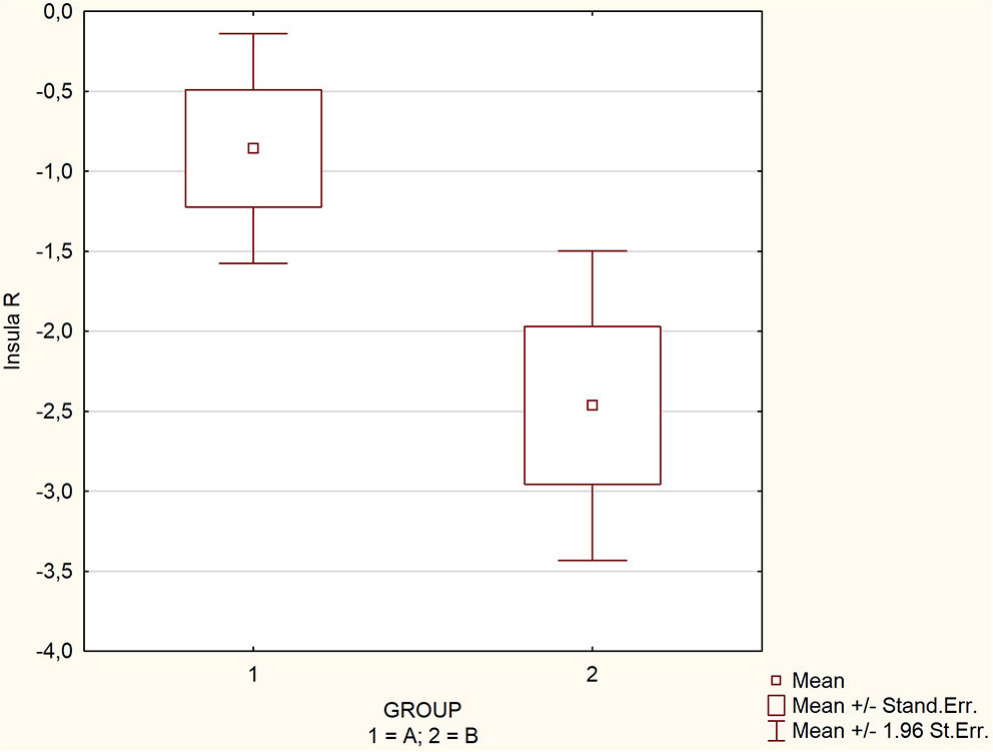
Comparison of perfusion in regions significantly affected by glycemic variability.

The mean, maximal, minimal, and standard deviation with 95% confidence interval values for the assessed parameters are listed in Table 1 for the whole group and subgroups. There were significant differences in the mean values of SPECT perfusion for two regions: hippocampus left (−1.2 vs. −2.8; *p* = 0.0086) and right (−0.8 vs. −2.1; *p* = 0.0075), and insula right (−0.9 vs. −2.5; *p* = 0.0035) (Table 1). The perfusion abnormalities in the group affected by glycemic variability in the other regions of interest were not significant. Spearman correlation coefficient showed an average negative correlation for the above-mentioned regions: hippocampus left, *Rs* = −0.48; hippocampus right *Rs* = −0.46; insula right, *Rs* = −0.47; pons, *Rs* = −0.47; thalamus left, *Rs* = −0.49 and whole brain, *Rs* = −0.42 (all Rs values were statistically significant *p* < 0.005, Table 2) (Figures 1–3).

**Table 1:** Basic statistics with subgroup comparison.

	**Whole group (*****N*** **=** **26)**		**Group A (*****N*** **=** **12)**		**Group B (*****N*** **=** **14)**		
	**Mean (Min-Max)**	**SD ± 95% CI**	**Mean (Min-Max)**	**SD ± 95% CI**	**Mean (Min-Max)**	**SD ± 95% CI**	**P**
III Ventricle (mm)	11.2 (6–17)	2.53 (1.98–3.49)	10.4 (6–14)	1.98 (1.4–3.35)	11.9 (7–17)	2.82 (2.05–4.55)	0.2368
PONS (cm2)	4.5 (3.3–5.8)	0.61 (0.47–0.84)	4.4 (3.3–5.8)	0.71 (0.5–0.5)	4.7 (3.8–5.6)	0.5 (0.36–0.8)	0.2801
MIDBRAIN (cm2)	0.8 (0.4–1.3)	0.26 (0.2–0.35)	0.8 (0.4–1.3)	0.24 (0.17–0.17)	0.8 (0.4–1.3)	0.28 (0.2–0.44)	0.607
P/M ratio	0.2 (0.1–0.3)	0.05 (0.04–0.07)	0.2 (0.1–0.3)	0.06 (0.04–0.04)	0.2 (0.1–0.2)	0.04 (0.03–0.07)	0.3681
Amygdala L	−1.4 (−4.2–2.7)	1.66 (1.3–2.3)	−1.1 (−4.2–2.7)	1.92 (1.36–1.36)	−1.6 (−3.1–1.5)	1.43 (1.04–2.31)	0.4253
Amygdala R	−1.1 (−3.8–1.3)	1.44 (1.13–1.99)	−0.6 (−2.6–1.3)	1.28 (0.91–0.91)	−1.6 (−3.8–1.3)	1.46 (1.06–2.35)	0.0896
Basal Ganglia L	−2.4 (−5.8–0.5)	1.92 (1.5–2.65)	−1.7 (−5.3–0.5)	1.7 (1.2–1.2)	−3 (−5.8–0.1)	1.94 (1.41–3.13)	0.076
Basal Ganglia R	−2.1 (−6.1–1.1)	2.05 (1.61–2.84)	−1.3 (−5.3–0.9)	1.82 (1.29–1.29)	−2.8 (−6.1–1.1)	2.07 (1.5–3.33)	0.0946
Brainstem	−2.9 (−7–1.8)	1.93 (1.51–2.69)	−2.3 (−5.3 to −0.3)	1.35 (0.94–0.94)	−3.4 (−7–1.8)	2.21 (1.6–3.56)	0.0798
Cerebellum L	−1.3 (−5.4–2.1)	2.06 (1.62–2.85)	−1.2 (−1.2–0)	1.23 (0.87–0.87)	−1.4 (−5.4–2.1)	2.62 (1.9–4.22)	0.817
Cerebellum R	−1.5 (−9.2–1.9)	2.49 (1.95–3.44)	−1.4 (−1.4–1.9)	3.11 (2.2–2.2)	−1.6 (−4.8–1.4)	1.93 (1.4–3.12)	0.4715
Frontal Lobe L	−1.4 (−6.9–2.6)	2.06 (1.62–2.84)	−1.1 (−1.1–1.8)	1.91 (1.35–1.35)	−1.7 (−6.9–2.6)	2.21 (1.6–3.55)	0.5371
Frontal Lobe R	−1.5 (−5.7–3.1)	1.96 (1.54–2.71)	−1.3 (−1.3–3.1)	2.01 (1.43–1.43)	−1.7 (−5.7–1.9)	1.98 (1.44–3.19)	0.959
Hippocampus L	−2.1 (−5.1–1.8)	1.75 (1.38–2.4)	−1.2 (−1.2–1.8)	1.77 (1.25–1.25)	−2.8 (−5.1 to −0.8)	1.45 (1.05–2.34)	0.0086
Hippocampus R	−1.5 (−4.8–1.4)	1.66 (1.3–2.29)	−0.8 (−0.8–1.4)	1.76 (1.25–1.25)	−2.1 (−4.1 to −0.2)	1.36 (0.99–2.19)	0.0075
Insula L	−3 (−7.1–2.4)	2.58 (2.03–3.54)	−2.1 (−2.1–2.4)	2.59 (1.84–1.84)	−3.9 (−7–0.4)	2.44 (1.77–3.94)	0.0803
Insula R	−1.7 (−6.2–1)	1.77 (1.39–2.45)	−0.9 (−0.9–1)	1.27 (0.9–0.9)	−2.5 (−6.2–1)	1.85 (1.34–2.98)	0.0035
Pons	−2.6 (−5–1.9)	1.28 (1.01–1.77)	−2.3 (−2.3 to −0.8)	0.76 (0.53–0.53)	−2.9 (−5–1.9)	1.59 (1.15–2.57)	0.0231
Temporal L	0.1 (−3–3.1)	1.51 (1.18–2.08)	0 (0–2.4)	1.36 (0.96–0.96)	0.3 (−3–3.1)	1.66 (1.2–2.67)	0.5542
Temporal R	1.4 (−4.1–4)	1.7 (1.34–2.34)	1.8 (1.8–3.2)	1.17 (0.83–0.83)	1.2 (−4.1–4)	2.12 (1.53–3.41)	0.5371
Thalamus L	−4.1 (−7 to −0.7)	1.83 (1.44–2.5)	−3.3 (−3.3 to −0.7)	1.55 (1.09–1.09)	−5 (−7 to −1.1)	1.76 (1.27–2.83)	0.0146
Thalamus R	−4.6 (−9.5 to −1.1)	2.22 (1.75–3.05)	−3.8 (−3.8 to −1.1)	2.39 (1.69–1.69)	−5.3 (−9.5 to −2.2)	1.99 (1.44–3.21)	0.0946
Whole Brain	−2.3 (−7.9–1.5)	2.22 (1.74–3.06)	−1.3 (−1.3–1.5)	1.9 (1.33–1.33)	−3.2 (−7.9–0.9)	2.24 (1.62–3.6)	0.0428

*Min, minimal value; Max, maximal value; SD, standard deviation; 95% CI, 95% confidence interval; p, p value for U Mann-Whitney test; in red are marked statistically significant p values*.

**Table 2:** Spearman correlation.

	**Rs**
III Ventricle (mm)	0.25
PONS (cm^2^)	0.22
MIDBRAIN (cm^2^)	−0.11
P/M ratio	−0.19
Amygdala L	−0.16
Amygdala R	−0.34
Basal Ganglia L	−0.36
Basal Ganglia R	−0.34
Brainstem	−0.36
Cerebellum L	0.05
Cerebellum R	−0.15
Frontal Lobe L	−0.13
Frontal Lobe R	−0.02
Hippocampus L	−0.48
Hippocampus R	−0.46
Insula L	−0.36
Insula R	−0.47
Pons	−0.47
Temporal L	0.12
Temporal R	−0.13
Thalamus L	−0.49
Thalamus R	−0.34
Whole Brain	−0.42

*Rs, Spearman correlation coefficient; in red are marked statistically significant values of Rs*.

## Discussion

### Hippocampus Hypoperfusion in Tauopathic Atypical Parkinsonian Syndrome

This study stresses the necessity of evaluating glycemic levels below the boundary of diabetes in neurodegenerative disorders. PSP and CBS are not fully recognized in the context of pathogenesis. The presented preliminary research should be considered as a factor in further discussions concerning deterioration within brain structures that may impact cognitive abilities, such as the hippocampus hypoperfusion observed in patients affected by glycemic variability. In our study, the results show that glycemic variability most significantly impacts certain regions of the brain, such as the hippocampus and insula. Moreover, there is a negative correlation between higher prediabetes glucose levels and SPECT-based perfusion images.

### Glycemic Variability and Prediabetes in Neurodegenerative Disorders

Prediabetes and glycemic variability are better known for their impact on the peripheral nervous system, however, recent papers have revealed interesting findings concerning the impact of prediabetes on brain amyloid accumulation and cognitive abilities (20, 21). The majority of the described reports discuss diseases other than PSP/CBS, however, they may be useful in understanding the underlying mechanisms that are likely also present in atypical tauopathic parkinsonian syndromes. In one study, the authors described the association between prediabetes and beta-amyloid concentration in late middle age. The same correlation was not confirmed in DM2. The authors of the study hypothesized that this may be related to the efficiency of diabetes treatment on brain beta-amyloid accumulation (20). Another study concerning the impact of prediabetes on the brain was performed on patients with Alzheimer's disease; the authors did not find any significant association between prediabetes and various analyzed factors such as hippocampal/intracranial volume ratio and cerebrospinal fluid phosphorylated tau-_181_/amyloid-β_1−42_ ratio (22). On the other hand, cognition was affected by abnormalities in brain metabolism. The metabolic evaluations were based on examinations using fluorodeoxyglucose in positron emission tomography. In a different study, prediabetes was associated with insulin resistance rather than solely based on the elevation of glucose levels in the context of cognitive deterioration (21). The literature concerning the correlation between prediabetes and brain abnormalities is limited. However, it has been demonstrated that in obese subjects with prediabetes the activation of reward-related areas is reduced significantly (23). In individuals with prediabetes, an increase in insulin resistance is associated with a decrease in the brain glucose metabolism rate in the frontal, parietotemporal, and cingulate gyrus regions (24). Zborowski et al. observed downregulation of hippocampal insulin and brain-derived neurotrophic factor signaling in prediabetic mice. On the other hand, a study by Soares al. based on increased hippocampal levels of AMPA and NMDA receptor subunits GluA1 and GLUN1 and decreased hippocampal glucocorticoid receptor levels, suggested that alteration of glutamatergic neurotransmission and abnormal glucocorticoid signaling in hippocampus is typical for prediabetes (25, 26). In a different study, the hippocampal region, as well as the whole brain, was associated with increased lactate production in prediabetes (27). There is also some data available on the volume of certain brain regions in prediabetes. Left hippocampal tail volume was interpreted as an early diabetes-related marker due to brain damage and a bilateral lateral hippocampus, left amygdala, and right putamen reduction in gray matter volume in prediabetes when compared to healthy controls (28, 29). Smaller hippocampal volumes and greater frontal lobe atrophy were typical for adolescents with insulin resistance (30). White matter changes, represented by alterations in the white matter micro-integrity of the anterior thalamic radiation and inferior and superior longitudinal fasciculi, are described as typical early signs of prediabetic brain (31). Reitz observed that abnormalities in glycaemia are associated with a higher number of brain infarcts, white matter hyperintensity volume, decreased total white matter and gray matter, and hippocampus volume (32). Nevertheless, a study by Schneider et al. indicated that there is no evidence that associations of diabetes with smaller brain volumes are mediated by brain vascular pathology (33). On the other hand, cerebrovascular impairment, which is present in prediabetes, may lead to a mild hypoxic state that, when accompanied by the metabolic dysfunction-driven suppression of neuronal autophagy, causes cognitive decline (34).

Though effects on the hippocampus are more commonly associated with Alzheimer's disease, considerable tau accumulation can be observed in the hippocampus in PSP; however, it was found to be independent and the tau accumulation in PSP is more pronounced in strategic regions related to the disease's progression such as the brainstem and basal ganglia (35). This finding could be analyzed in the context of an association between tau accumulation and microglial activation in PSP. Microglial activation, on the other hand, is also associated with diabetes and other glycemic disturbances. This series of possible mechanisms leading to hippocampus vulnerability have not been fully recognized.

Observations concerning abnormal transglutaminase activity in PSP as well as other tauopathies were described in previous research; however, no abnormal transglutaminase-induced crosslinking activity with glycemic variability was found in PSP and CBS (36, 37). Though transglutaminase is associated with an indirect impact on neurodegeneration, the enzyme is considered to be a factor leading to the neuropathology after the initiation of the disease process. Glycemic variability, though not proven to be associated with transglutaminase's impact on neurodegeneration, appears to act as an enhancer of clinical decline and structural hypoperfusion; however, insufficient data in the field make any interpretation questionable and requiring further verification.

### Clinical Significance

The possible disturbance of hippocampal function is commonly associated with cognitive decline. Recent studies showed associations between tau pathology and clinical symptoms in the subfield of hippocampal formation and indicated associations with executive function and behavior (38). The significance of the deterioration within the hippocampus in PSP/CBS was presented in various studies. The features possibly affected by this disturbance include multiple speech and language domains (39). The literature concerning the impact of hippocampal deterioration and its clinical significance in PSP/CBS is limited. The most striking issue highlighted in this study is undoubtedly related to the possible reversibility of mechanisms leading to hippocampal dysfunction, as glycemic variability appears to be a modifiable risk factor.

### Limitations

This is a small pilot study; however, it stresses the significance of glycemic variability as a risk factor for further deterioration in tauopathic atypical parkinsonian syndromes. The study was limited to tauopathic atypical parkinsonian syndromes due to their rarity, difficulties in examination, and the fact that they are underdiagnosed in the context of glycemic variability, as only a few studies highlight the issue of diabetes in PSP. To the best of our knowledge, no such study has been conducted in prediabetes and glycemic variability groups. The authors did not perform an additional examination of SPECT in subjects with increased glycemic variability without neurodegenerative disorders. This is related to the preliminary character of the study and to the fact that the aim of the study is to highlight the significance of glucose metabolism impairment as a possible enhancer of deterioration rate in tauopathic parkinsonian syndromes. Additionally, due to the pilot character of the study, the authors were not granted permission by the Ethical Committee to use radiotracers in patients without neurodegenerative disease in this study considering the availability of a software database of healthy volunteers age-matched with the examined group of PSP/CBS patients. The authors did not create separate groups for PSP and CBS patients, as previous studies showed that perfusion assessment does not show significant differences in the differential diagnoses of these diseases (6). Due to the fact that all included patients are alive, no neuropathological verification could be conducted.

## Conclusion

The study results stress the necessity of early diagnosis of glycemic variability in neurodegenerative diseases. The significant decrease in perfusion of the hippocampus in patients with PSP/CBS is an interesting observation considering the diverse course of cognitive deterioration among patients with PSP and CBS. The results of the study should be interpreted as a factor encouraging discussion of hippocampal vulnerability to glycemic variability and its possible links with pathomechanisms of neurodegeneration. Early initiation of treatment and control of glycemic variability and prediabetes may have a beneficial role that impacts the rate of deterioration of cognitive impairment. Further research in the field on the role of glycemic variability and prediabetes in tauopathic atypical parkinsonism is required.

## Data Availability Statement

The original contributions presented in the study are included in the article/supplementary material, further inquiries can be directed to the corresponding author/s.

## Ethics Statement

The studies involving human participants were reviewed and approved by Ethical Committee of Medical University of Warsaw–AKBE243/ 2016. The patients/participants provided their written informed consent to participate in this study.

## Author Contributions

PA and NM: study design, manuscript preparation, and data collection. AD and LK: manuscript preparation. BM: manuscript preparation and statistical analysis. All authors contributed to the article and approved the submitted version.

## Funding

Internal funds were provided by the Department of Neurology.

## Conflict of Interest

The authors declare that the research was conducted in the absence of any commercial or financial relationships that could be construed as a potential conflict of interest.

## Publisher's Note

All claims expressed in this article are solely those of the authors and do not necessarily represent those of their affiliated organizations, or those of the publisher, the editors and the reviewers. Any product that may be evaluated in this article, or claim that may be made by its manufacturer, is not guaranteed or endorsed by the publisher.

## References

[1] Höglinger GU Respondek G Stamelou M Kurz C Josephs KA Lang AE. Movement disorder society-endorsed PSP study group. Clinical diagnosis of progressive supranuclear palsy: the movement disorder society criteria. Mov Disord. (2017) 32:853–64. 10.1002/mds.26987 28467028 PMC5516529

[2] Armstrong MJ Litvan I Lang AE Bak TH Bhatia KP Borroni B. Criteria for the diagnosis of corticobasal degeneration. Neurology. (2013) 80:496–503. 10.1212/WNL.0b013e31827f0fd1 23359374 PMC3590050

[3] Jabbari E Holland N Chelban V Jones PS Lamb R Rawlinson C. Diagnosis across the spectrum of progressive supranuclear palsy and corticobasal syndrome. JAMA Neurol. (2020) 77:377–87. 10.1001/jamaneurol.2019.4347 31860007 PMC6990759

[4] Alster P Krzyzanowska E Koziorowski D Szlufik S Rózański D Noskowska J. Difficulties in the diagnosis of four repeats (4R) tauopathic parkinsonian syndromes. Neurol Neurochir Pol. (2018) 52:459–64. 10.1016/j.pjnns.2018.06.002 30025721

[5] Respondek G Grimm MJ Piot I Arzberger T Compta Y Englund E. Movement disorder society-endorsed progressive supranuclear palsy study group. Validation of the movement disorder society criteria for the diagnosis of 4-repeat tauopathies. Mov Disord. (2020) 35:171–6. 10.1002/mds.27872 31571273 PMC7993399

[6] Alster P Nieciecki M Koziorowski D Cacko A Charzyńska I Królicki L. Is brain perfusion a differentiating feature in the comparison of Progressive Supranuclear Palsy Syndrome (PSPS) and Corticobasal Syndrome (CBS)? J Clin Neurosci. (2020) 77:123–7. 10.1016/j.jocn.2020.05.005 32389545

[7] Rabadia SV Litvan I Juncos J Bordelon Y Riley DE Standaert D. Hypertension and progressive supranuclear palsy. Parkinsonism Relat Disord. (2019) 66:166–70. 10.1016/j.parkreldis.2019.07.036 31420308

[8] Kwasny MJ Oleske DM Zamudio J Diegidio R Höglinger GU. Clinical features observed in general practice associated with the subsequent diagnosis of progressive supranuclear palsy. Front Neurol. (2021) 12:637176. 10.3389/fneur.2021.637176 33967937 PMC8100604

[9] Koga S Roemer SF Kasanuki K Dickson DW. Cerebrovascular pathology presenting as corticobasal syndrome: an autopsy case series of “vascular CBS”. Parkinsonism Relat Disord. (2019) 68 79–84. 10.1016/j.parkreldis.2019.09.001 31621626 PMC7141792

[10] Josephs KA Ishizawa T Tsuboi Y Cookson N Dickson DW. A clinicopathological study of vascular progressive supranuclear palsy: a multiinfarct disorder presenting as progressive supranuclear palsy. Arch Neurol. (2002) 59:1597–601. 10.1001/archneur.59.10.1597 12374498

[11] Mccrimmon RJ. Consequences of recurrent hypoglycaemia on brain function in diabetes. Diabetologia. (2021) 64:971-7. 10.1007/s00125-020-05369-0 33738528 PMC8012314

[12] Giordani I Di Flaviani A Picconi F Malandrucco I Ylli D Palazzo P. Acute hyperglycemia reduces cerebrovascular reactivity: the role of glycemic variability. J Clin Endocrinol Metab. (2014) 99:2854–60. 10.1210/jc.2014-1087 24878046

[13] Cui X Abduljalil A Manor BD Peng CK Novak V. Multi-scale glycemic variability: a link to gray matter atrophy and cognitive decline in type 2 diabetes. PLoS ONE. (2014) 9:e86284. 10.1371/journal.pone.0086284 24475100 PMC3901681

[14] Hui J Zhang J Mao X Li Z Li X Wang F. The initial glycemic variability is associated with early neurological deterioration in diabetic patients with acute ischemic stroke. Neurol Sci. (2018) 39:1571–7. 10.1007/s10072-018-3463-6 29869743

[15] Lim JS Kim C Oh MS Lee JH Jung S Jang MU. Effects of glycemic variability and hyperglycemia in acute ischemic stroke on post-stroke cognitive impairments. J Diabetes Complications. (2018) 32:682–7. 10.1016/j.jdiacomp.2018.02.006 29793824

[16] Yoon JE Sunwoo JS Kim JS Roh H Ahn MY Woo HY. Poststroke glycemic variability increased recurrent cardiovascular events in diabetic patients. J Diabetes Complications. (2017) 31:390–4. 10.1016/j.jdiacomp.2016.11.014 27956053

[17] Cai Y Wang C Di W Li W Liu J Zhou S. Correlation between blood glucose variability and the risk of death in patients with severe acute stroke. Rev Neurol (Paris). (2020) 176:582–6. 10.1016/j.neurol.2019.12.003 31911002

[18] Livny A Ravona-Springer R Heymann A Priess R Kushnir T Tsarfaty G. Long-term variability in glycemic control is associated with white matter hyperintensities in APOE4 genotype carriers with type 2 diabetes. Diabetes Care. (2016) 39:1056–9. 10.2337/dc15-2331 27208321 PMC5317241

[19] Matsushima K Peng M Velasco C Schaefer E Diaz-Arrastia R Frankel H. Glucose variability negatively impacts long-term functional outcome in patients with traumatic brain injury. J Crit Care. (2012) 27:125–31. 10.1016/j.jcrc.2011.08.012 22033047

[20] Luchsinger JA Palta P Rippon B Sherwood G Soto L Ceballos F. Pre-diabetes, but not type 2 diabetes, is related to brain amyloid in late middle-age. J Alzheimers Dis. (2020) 75:1241–52. 10.3233/JAD-200232 32390636 PMC7659021

[21] Willmann C Brockmann K Wagner R Kullmann S Preissl H Schnauder G. Insulin sensitivity predicts cognitive decline in individuals with prediabetes. BMJ Open Diabetes Res Care. (2020) 8:e001741. 10.1136/bmjdrc-2020-001741 33203727 PMC7674089

[22] Sundermann EE Thomas KR Bangen KJ Weigand AJ Eppig JS Edmonds EC. Prediabetes is associated with brain hypometabolism and cognitive decline in a sex-dependent manner: a longitudinal study of nondemented older adults. Front Neurol. (2021) 12:551975. 10.3389/fneur.2021.551975 33679574 PMC7933503

[23] Farr OM Mantzoros CS. Obese individuals with more components of the metabolic syndrome and/or prediabetes demonstrate decreased activation of reward-related brain centers in response to food cues in both the fed and fasting states: a preliminary fMRI study. Int J Obes (Lond). (2017) 41:471–4. 10.1038/ijo.2016.231 28017966 PMC5340581

[24] Baker LD Cross DJ Minoshima S Belongia D Watson GS Craft S. Insulin resistance and Alzheimer-like reductions in regional cerebral glucose metabolism for cognitively normal adults with prediabetes or early type 2 diabetes. Arch Neurol. (2011) 68:51-7. 10.1001/archneurol.2010.225 20837822 PMC3023149

[25] Zborowski VA Heck SO Marques LS Bastos NK Nogueira CW. Memory impairment and depressive-like phenotype are accompanied by downregulation of hippocampal insulin and BDNF signaling pathways in prediabetic mice. Physiol Behav. (2021) 237:113346. 10.1016/j.physbeh.2021.113346 33545209

[26] Soares E Prediger RD Nunes S Castro AA Viana SD Lemos C. Spatial memory impairments in a prediabetic rat model. Neuroscience. (2013) 250:565–77. 10.1016/j.neuroscience.2013.07.055 23912035

[27] Choi YS Song JE Lee JE Kim E Kim CH Kim DH. Hyperpolarized [1-13C]lactate flux increased in the hippocampal region in diabetic mice. Mol Brain. (2019) 12:88. 10.1186/s13041-019-0505-9 31675964 PMC6824044

[28] Dong S Dongwei L Zhang J Liang J Sun Z Fang J. Individuals in the prediabetes stage exhibit reduced hippocampal tail volume and executive dysfunction. Brain Behav. (2019) 9:e01351. 10.1002/brb3.1351 31240857 PMC6710206

[29] Cui D Liu X Liu M Cao W Xue Y Guo Y. Subcortical gray matter structural alterations in prediabetes and type 2 diabetes. Neuroreport. (2019) 30:441–5. 10.1097/WNR.0000000000001224 30855559

[30] Ursache A Wedin W Tirsi A Convit A. Preliminary evidence for obesity and elevations in fasting insulin mediating associations between cortisol awakening response and hippocampal volumes and frontal atrophy. Psychoneuroendocrinology. (2012) 37:1270–6. 10.1016/j.psyneuen.2011.12.020 22265870 PMC3337891

[31] Hou YC Yang SH Wu YT Lai CH. Alterations of neocortico-limbic association fibers and correlation with diet in prediabetes diagnosed by impaired fasting glucose. J Magn Reson Imaging. (2016) 43:1500–6. 10.1002/jmri.25127 26756544

[32] Reitz C Guzman VA Narkhede A Decarli C Brickman AM Luchsinger JA. Relation of dysglycemia to structural brain changes in a multiethnic elderly cohort. J Am Geriatr Soc. (2017) 65:277–85. 10.1111/jgs.14551 27917464 PMC5311018

[33] Schneider ALC Selvin E Sharrett AR Griswold M Coresh J Jack CR. Diabetes, prediabetes, and brain volumes and subclinical cerebrovascular disease on MRI: the Atherosclerosis Risk in Communities Neurocognitive Study (ARIC-NCS). Diabetes Care. (2017) 40:1514–21. 10.2337/dc17-1185 28916531 PMC5652590

[34] Fakih W Mroueh A Salah H Eid AH Obeid M Kobeissy F. Dysfunctional cerebrovascular tone contributes to cognitive impairment in a non-obese rat model of prediabetic challenge: role of suppression of autophagy and modulation by anti-diabetic drugs. Biochem Pharmacol. (2020) 178:114041. 10.1016/j.bcp.2020.114041 32439335

[35] Kovacs GG Lukic MJ Irwin DJ Arzberger T Respondek G Lee EB. Distribution patterns of tau pathology in progressive supranuclear palsy. Acta Neuropathol. (2020) 140:99–119. 10.1007/s00401-020-02158-2 32383020 PMC7360645

[36] Jeitner TM Pinto JT Krasnikov BF Horswill M Cooper AJ. Transglutaminases and neurodegeneration. J Neurochem. (2009) 109(Suppl. 1):160–6. 10.1111/j.1471-4159.2009.05843.x 19393023 PMC2752967

[37] Marsili L Sharma J Espay AJ Migazzi A Abdelghany E Hill EJ. Neither a novel tau proteinopathy nor an expansion of a phenotype: reappraising clinicopathology-based nosology. Int J Mol Sci. (2021) 22:7292. 10.3390/ijms22147292 34298918 PMC8329925

[38] Ge X Zhang D Qiao Y Zhang J Xu J Zheng Y. Association of Tau pathology with clinical symptoms in the subfields of hippocampal formation. Front Aging Neurosci. (2021) 13:672077. 10.3389/fnagi.2021.672077 34335226 PMC8317580

[39] Peterson KA Jones PS Patel N Tsvetanov KA Ingram R Cappa SF. Language disorder in progressive supranuclear palsy and corticobasal syndrome: neural correlates and detection by the MLSE screening tool. Front Aging Neurosci. (2021) 13:675739. 10.3389/fnagi.2021.675739 34381350 PMC8351757

